# Baicalin Protects Mice from Aristolochic Acid I-Induced Kidney Injury by Induction of CYP1A through the Aromatic Hydrocarbon Receptor

**DOI:** 10.3390/ijms160716454

**Published:** 2015-07-20

**Authors:** Ke Wang, Chenchen Feng, Chenggang Li, Jun Yao, Xiaofeng Xie, Likun Gong, Yang Luan, Guozhen Xing, Xue Zhu, Xinming Qi, Jin Ren

**Affiliations:** 1 Key Laboratory of Nuclear Medicine, Ministry of Health, Jiangsu Key Laboratory of Molecular Nuclear Medicine, Jiangsu Institute of Nuclear Medicine, Wuxi 214063, Jiangsu, China; E-Mails: wangke@jsinm.org (K.W.); zhuxue@jsinm.org (X.Z.); 2 Center for Drug Safety Evaluation and Research, Shanghai Institute of Materia Medica, Chinese Academy of Sciences, Graduate School of the Chinese Academy of Sciences, Shanghai 201203, China; E-Mails: shang320@hotmail.com (C.F.); lifen_888@hotmail.com (C.L.); jyao@cdser.simm.ac.cn (J.Y.); cluseee@gmail.com (X.X.); lkgong@cdser.simm.ac.cn (L.G.); djlover@gmail.com (Y.L.); gzxing@cdser.simm.ac.cn (G.X.)

**Keywords:** aristolochic acid, kidney injury, baicalin, aromatic hydrocarbon receptor, CYP1A

## Abstract

Exposure to aristolochic acid I (AAI) can lead to aristolochic acid nephropathy (AAN), Balkan endemic nephropathy (BEN) and urothelial cancer. The induction of hepatic CYP1A, especially CYP1A2, was considered to detoxify AAI so as to reduce its nephrotoxicity. We previously found that baicalin had the strong ability to induce CYP1A2 expression; therefore in this study, we examined the effects of baicalin on AAI toxicity, metabolism and disposition, as well as investigated the underlying mechanisms. Our toxicological studies showed that baicalin reduced the levels of blood urea nitrogen (BUN) and creatinine (CRE) in AAI-treated mice and attenuated renal injury induced by AAI. Pharmacokinetic analysis demonstrated that baicalin markedly decreased AUC of AAI in plasma and the content of AAI in liver and kidney. CYP1A induction assays showed that baicalin exposure significantly increased the hepatic expression of CYP1A1/2, which was completely abolished by inhibitors of the Aromatic hydrocarbon receptor (AhR), 3ʹ,4ʹ-dimethoxyflavone and resveratrol, *in vitro* and *in vivo*, respectively. Moreover, the luciferase assays revealed that baicalin significantly increased the luciferase activity of the reporter gene incorporated with the Xenobiotic response elements recognized by AhR. In summary, baicalin significantly reduced the disposition of AAI and ameliorated AAI-induced kidney toxicity through AhR-dependent CYP1A1/2 induction in the liver.

## 1. Introduction

Herbal drugs derived from *Aristolochia* species have been used for the treatment of arthritis, gout, rheumatism and festering since antiquity [1]. Aristolochic acid (AA) is the active component of *Aristolochia* species, consisting of a mixture of structurally related nitrophenanthrene carboxylic acids, mainly aristolochic acid I (AAI) and aristolochic acid II (AAII) [2]. AA was used worldwide for a long time due to its anti-inflammatory properties, until the first case of nephropathy was reported in Belgium, which is now known as aristolochic acid nephropathy (AAN) [3]. More recently, exposure to AA has also been involved with Balkan endemic nephropathy (BEN) and its associated urothelial cancer [4]. However, *Aristolochia* plants containing AA are still being used as traditional medicines in some parts of the world [5].

In studying AAI-induced toxicity in humans, it is of major importance to elucidate the activation mechanisms of AAI, the major nephrotoxic constituent of AA. We previously demonstrated that AAI-induced nephrotoxicity was more severe when liver-specific NAPDH-cytochrome P450 reductase (CPR) was deficient [6,7], and the induction of CYP1A significantly reduced AAI-induced kidney toxicity in wild-type mice [8,9]. 3-Methylcholanthrene (3-MC) and β-naphthoflavone (BNF) are the known inducers of CYP1A [10,11,12]; however, their applications were largely limited due to their genotoxicity [13,14]. Therefore, safer drugs are required for the prevention or treatment of AAI-induced toxicity.

In Asia, AAI is always prescribed in adjunct with other herbs including *Scutellaria baicalensis*, licorice root and *Radix Puerariae* by herbalists [15,16]. Therefore, concomitant use of herbal compounds targeting CYP1A may be beneficial for the attenuation of AAI-induced toxicity. In our study, various herbal compounds were screened for their capabilities in inducing CYP1A and baicalin, a type of flavonoid, was shown to be the most potent inducer of CYP1A1/2, especially CYP1A2. In the current study, we examined the effects of baicalin on the toxicity, metabolism and disposition of AAI as well as investigated the mechanism through which, baicalin induced CYP1A1/2 in mouse liver.

## 2. Results and Discussion

### 2.1. Results

#### 2.1.1. Screening of Herbal Compounds with CYP1A2 Induction Assays

**Figure 1:**
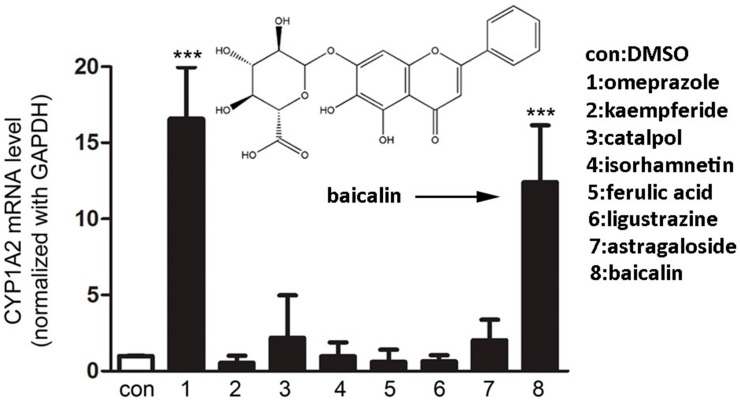
Screening of herbal compounds for their activities in inducing CYP1A2 in Fa2N-4 cells. Cells were pre-treated with candidate compounds at 10 μM. *CYP1A2* mRNA was quantified by real-time PCR. con: control (negative control); 1: omeprazole (positive control); 2: kaempferide; 3: catalpol; 4: isorhamnetin; 5: ferulic acid; 6: ligustrazine; 7: astragaloside; 8: baicalin. *** *p* < 0.001 *versus* the negative control.

To screen CYP1A2 inducers, Fa2N-4 cells, a non-tumorigenic immortalized human hepatic cell line, were treated with seven different herbal compounds. The results showed that baicalin is the most potent compound in inducing *CYP1A2* gene expression at the mRNA level (Figure 1).

#### 2.1.2. Effects of Baicalin on Aristolochic Acid I (AAI)-Induced Renal Damage

**Figure 2:**
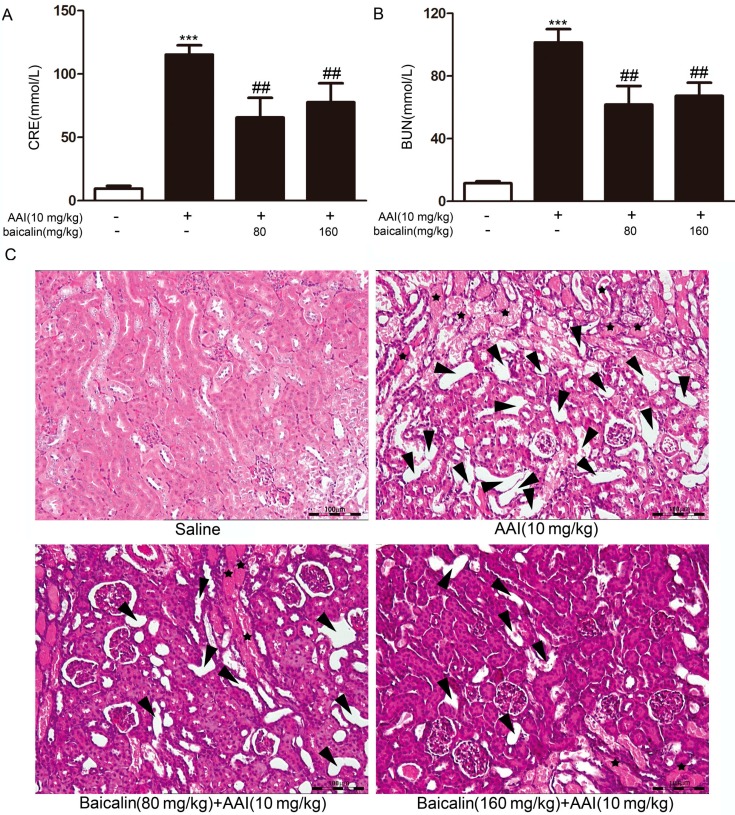
Effects of baicalin on aristolochic acid I (AAI) nephrotoxicity. Blood samples were collected to obtain serum for the measurement of blood urea nitrogen (BUN) (**A**) and creatinine (CRE) (**B**); (**C**) Kidneys were collected to perform hematoxylin and eosin (H&E) staining. Arrowheads, tubular dilation; stars, tubular necrosis and granular casts. Scale bar, 100 μm. Data are expressed as the mean ± SD (*n* = 5). *** *p* < 0.001 *versus* the control, ^##^ *p* < 0.01 *versus* the AAI only group.

Mice were pretreated with baicalin for three days. Baicalin pretreatment significantly reduced the levels of BUN and CRE induced by AAI (Figure 2A,B). Lesions were observed in the kidneys after AAI administration by histopathological examination. Lesions representing extensive tubular necrosis, and tubular dilation occurred at seven days after AAI administration in the AAI group. Kidneys from mice in the baicalin-pretreated group displayed fewer lesions (Figure 2C). Together, these results demonstrated that baicalin protected mice from AAI-induced renal damage.

#### 2.1.3. Effects of Baicalin on AAI metabolism in the Plasma

**Figure 3:**
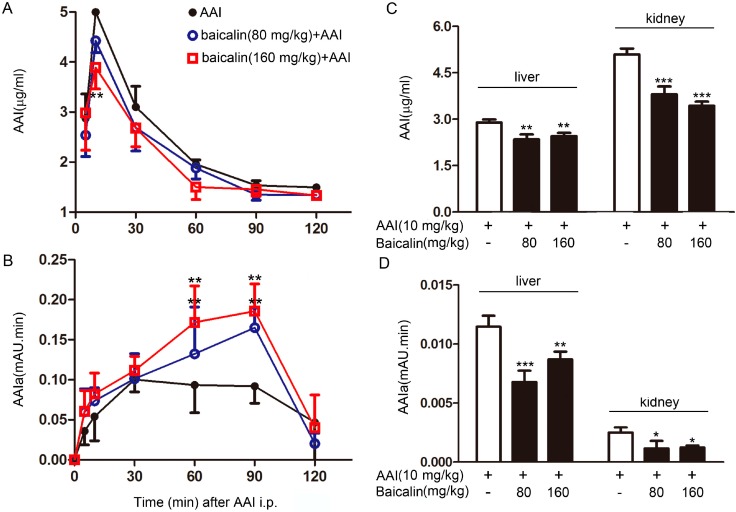
Levels of AAI and its major metabolite AAIa in the plasma, liver, and kidney. Blood samples were collected from mice at the indicated time points after AAI injection. Plasma levels of AAI (**A**) and AAIa (**B**) were measured by high-performance liquid chromatography (HPLC); Tissue samples from mice were collected at 30 min after AAI injection to determine the levels of AAI (**C**) and AAIa (**D**). Data are expressed as the mean ± SD (*n* = 5). * *p* < 0.05, ** *p* < 0.01, *** *p* < 0.001 *versus* the AAI group.

AAIa is the major metabolite of AAI oxidative metabolism by CYP1A. We studied the pharmacokinetics of AAI and its metabolites *in vivo*. Baicalin pretreatment markedly decreased the level of AAI in mice, as indicated by dramatically reduced pharmacokinetic parameters, such as the area under the curve (AUC) of AAI, in the baicalin + AAI group compared to those in the AAI group following a single intra-peritoneal (i.p.) dose of AAI at 10 mg/kg (Figure 3A, Table 1). Simultaneously, the level of AAIa, the major metabolite of AAI, was higher in the plasma of the baicalin-pretreated group than that in the AAI group (Figure 3B). These results suggested that baicalin pretreatment can accelerate the metabolism of AAI *in vivo*.

**Table 1:** Comparison of pharmacokinetic parameters between AAI and Baicalin + AAI treated mice.

Groups	*C*_max_ (μg/mL)	*T*_max_ (min)	AUC (min·μg/mL)	*t*_1/2_ (min)
AAI	4.99 ± 0.41	10.00 ± 0	844.96 ± 40.12	78.68 ± 12.88
Baicalin (80 mg/kg) + AAI	4.43 ± 0.24	10.00 ± 0	759.39 ± 23.26 **	77.96 ± 6.84
Baicalin (160 mg/kg) + AAI	3.89 ± 0.43	9 ± 2.24	710.07 ± 33.95 ***	80.83 ± 3.02

Values are expressed as the mean ± SD (*n* = 5); ** *p* < 0.01, *** *p* < 0.001 *versus* the AAI group.

#### 2.1.4. Effects of Baicalin on AAI Distribution in Tissues

To examine whether the change in pharmacokinetics of AAI upon baicalin pretreatment was due to changes in the tissue distribution of AAI, the levels of AAI and AAIa in the livers and kidneys were measured by HPLC. Thirty minutes after a single i.p. injection of AAI at 10 mg/kg, the level of AAI in the kidneys was found to be higher than that in the livers; while the level of AAIa exhibited the opposite distribution pattern. Baicalin pretreatment resulted in reduced levels of AAI and AAIa in both the livers and kidneys (Figure 3C,D).

#### 2.1.5. Mechanism Underpinning the Protective Effect of Baicalin against AA Injury

**Figure 4:**
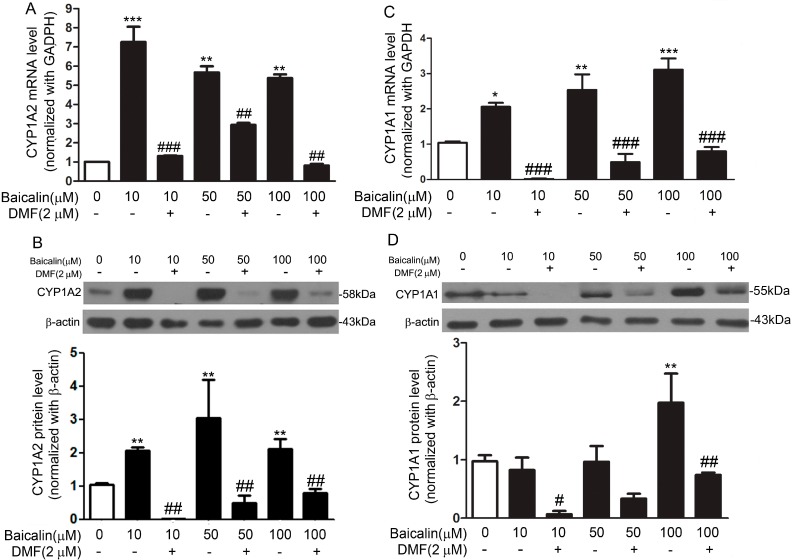
Baicalin increased CYP1A1 and CYP1A2 expression at the mRNA and protein level in Fa2N-4 cells. Cells were treated with baicalin (0–100 μM) for 24 h in the presence or absence of 3ʹ,4ʹ-dimethoxyflavone (DMF) (2 μM), a specific antagonist of AhR. mRNA level of *CYP1A2* was determined with real-time PCR and data were normalized to the expression of *GAPDH* (**A**); Protein expression of CYP1A2 was assessed through Western blot and data were normalized to that of β-actin (**B**); mRNA level of *CYP1A1* was determined with real-time PCR and data were normalized to the expression of *GAPDH* (**C**); Protein expression of CYP1A1 was assessed through Western blot and data were normalized to that of β-actin (**D**). Data are expressed as the mean ± SD (*n* = 5). * *p* < 0.05, ** *p* < 0.01, *** *p* < 0.001 *versus* the control group; ^#^ *p* < 0.05, ^##^ *p* < 0.01, ^###^ *p* < 0.001 *versus* the baicalin only group.

**Figure 5:**
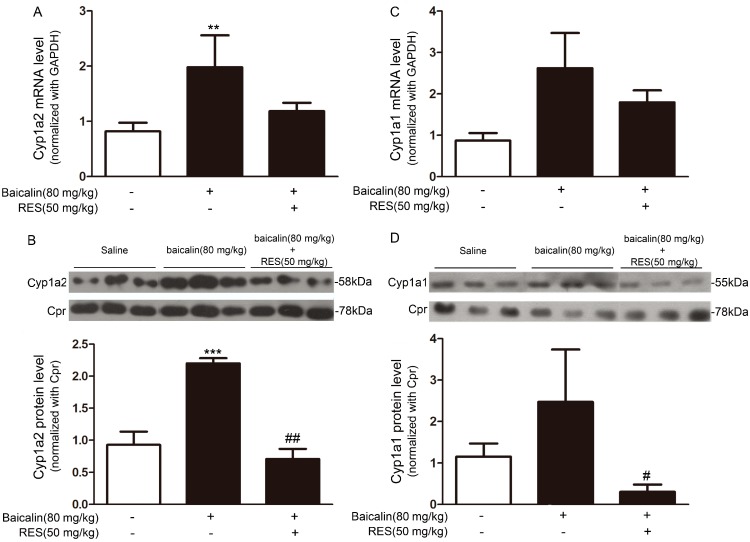
Baicalin increased the expression of CYP1A1 and CYP1A2 at the mRNA and protein level in mouse livers. Mice were treated with baicalin (80 mg/kg) for three days in the presence or absence of resveratrol (RES) (50 mg/kg), another specific antagonist of AhR. mRNA epressions of *CYP1A2* (**A**) and *CYP1A1* (**C**) were determined with real-time PCR and data were normalized to that of *GAPDH*; Liver microsomes were extracted and separated by SDS-PAGE. Protein expressions of CYP1A2 (**B**) and CYP1A1 (**D**) were assessed by Western blot and data were normalized to that of cytochrome P450 reductase (CPR). Data are expressed as the mean ± SD (*n* = 5). ** *p* < 0.01, *** *p* < 0.001 *versus* the control group; ^#^ *p* < 0.05, ^##^ *p* < 0.01, *versus* the baicalin only group.

To explore whether the protective effect of baicalin against AA nephrotoxicity was due to the induction of CYP1A in the liver, we analyzed the CYP1A expression upon baicalin treatment *in vitro* and *in vivo*. Fa2N-4 cells were treated with a range of concentrations of baicalin (0–100 μM) for 24 h. Baicalin increased CYP1A2 mRNA and protein expressions in a concentration-dependent manner (Figure 4A,B). CYP1A1 expression was also increased at both the mRNA (Figure 4C) and protein level after the treatment with 100 μM baicalin. And a relatively mild effect was observed in the cells treated with 10 or 50 μM baicalin (Figure 4D). In baicalin-pretreated mice, CYP1A1 (Figure 5A,B) and CYP1A2 (Figure 5C,D) expression were pronouncedly increased in the liver at the mRNA and protein level.

#### 2.1.6. Role of AhR in Baicalin-Induced CYP1A Induction

Mammalian *CYP1A* and *CYP1B* genes (encoding cytochrome P450 1A1, 1A2, and 1B1, respectively) are regulated mostly by AhR [17]. Upon ligand binding, AhR forms a heterodimer with the AhR nuclear translocator (ARNT) and the AhR-ARNT complex, which binds to specific XREs and activates *CYP1A* and *CYP1B* gene expression [18,19]. We used the AhR antagonists DMF and RES *in vitro* and *in vivo*, to evaluate whether the induction of CYP1A1/2 by baicalin was AhR-dependent. In Fa2N-4 cells, baicalin significantly increased the expression of CYP1A1/2 and such effect was abolished by the treatment of DMF (Figure 4). *In vivo*, RES pronouncedly attenuated the induction of hepatic CYP1A1/2 by baicalin (Figure 5).

**Figure 6:**
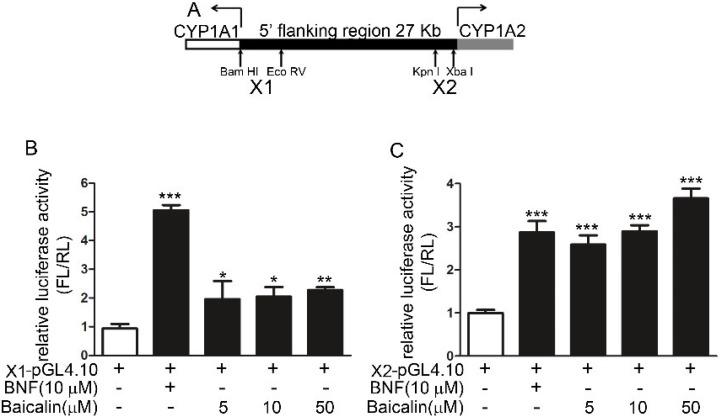
Effects of baicalin on the AhR-mediated activation of different segments of the *CYP1A* promoter in HepG2 cells. (**A**) Location of two insert segments, X1 and X2; and (**B**) Effects of baicalin and BNF on the luciferase activity of pGL4.10-X1; (**C**) Effects of baicalin and BNF on the luciferase activity of pGL4.10-X2. Luciferase activity was determined by firefly luciferase assay with data normalized to renilla luciferase activity. The results are expressed as the activity in drug treatment groups relative to that of the vehicle control. Each value is expressed as the mean ± SD. * *p* < 0.05, ** *p* < 0.01, *** *p* < 0.001 *versus* the plasmid only group.

Two pGL4.10 luciferase plasmids containing X1 (+1 to −2867 of CYP1A1) and X2 (−21,148 to −24,409 of CYP1A1; Figure 6A) segments were transfected into HepG2 cells to investigate the effects of baicalin. Treatment with either baicalin or BNF (positive control) increased the luciferase activity (Figure 6B,C), indicating the direct binding between the potential AhR complex and the CYP1A promoter.

### 2.2. Discussion

Human CYP1A1 and CYP1A2 are the most important enzymes involved in the biotransformation of AAI to AAIa [20,21]. Our findings showed that baicalin ameliorated AAI-induced renal toxicity via AhR-dependent induction of CYP1A. Supportive evidence includes: (1) baicalin attenuated the renal toxicity induced by AAI (Figure 2) through a significant decrease in AAI content in the kidneys after baicalin pretreatment (Figure 3); (2) we showed that baicalin significantly induces CYP1A1/2 expression in hepatocytes in both *in vitro* and *in vivo* conditions (Figure 4 and Figure 5); and (3) the AhR antagonists 3ʹ,4ʹ-DMF and RES reversed the effects of baicalin in the CYP1A1/2 induction. Our data indicated the important role of AhR in this process, which was further supported by the luciferase assays with reporter constructs containing the CYP1A promoter regions recognized by AhR (Figure 4, Figure 5 and Figure 6). Taken together, we demonstrated that AhR-mediated CYP1A induction is possibly responsible for the protective effects of baicalin against AA toxicity.

CYP1A2 showed much higher expression than CYP1A1 upon the treatment of baicalin (Figure 4 and Figure 5). CYP1A2 is the major CYP1A enzyme expressed in the livers of humans and mice. In addition, recent studies found that CYP1A2 has higher efficiency in the detoxification of AAI than CYP1A1 [22]. Therefore, it is plausible that CYP1A2 plays a more important role than CYP1A1 in the protective effects of baicalin against AA toxicity.

In the 5′-flanking regions of CYP1A1 and CYP1A2, there are several xenobiotic response element (XRE) segments, each of which has been shown to contribute differently to the chemical-induced gene transcription via segment deletion construct analysis. Previous reports showed that BNF, 3-MC and omeprazole have diverse effects on each XRE segment [23,24]. Our results indicated that the X2 segment may have higher sensitivity to baicalin, which may contribute predominantly to CYP1A2 induction by baicalin.

The formation of AAIa, the major metabolite of AAI detoxification, was expected to increase upon the induction of CYP1A catalysis in the livers. However, in our study, while AAI was decreased after baicalin pretreatment, AAIa was also decreased in the liver. The lack of increase in AAIa indicated that AAIa may have undergone a phase II conjugation, like UDP-glucuronosyltransferase (UGT), and is readily eliminated from the body [25]. As reported previously, UGT is activated through the AhR pathway, which is also involved in baicalin-induced CYP1A induction [26,27]. Thus, induction of UGT by baicalin may contribute to the decrease of AAIa in the liver.

Baicalin largely increased CYP1A expression in humanized cells and mouse livers. Moreover, CYP1A is highly conserved in humans and mice, implying that baicalin may exert similar effects in human. Although baicalin only results in a moderate reduction in AA-induced injury, it is clinically important as a non-toxic or carcinogenic compound [28,29] and therefore, may be a better candidate agent in the early prevention of AA-induced injury.

Overall, our study demonstrated that baicalin protected mice from AAI-induced renal injury through AhR-mediated CYP1A induction. Our study suggested that combined therapy of AA and herbal extracts, in particular baicalin may be clinically important. However, it is still necessary for healthcare providers to minimize the usage of herbal medicines containing AA.

## 3. Materials and Methods

### 3.1. Chemicals

Aristolochic acid I (AAI), dimethyl sulfoxide (DMSO) and 3ʹ,4ʹ-dimethoxyflavone (DMF) were purchased from Sigma (St. Louis, MO, USA). Baicalin and resveratrol (RES) were purchased from Zelang (Nanjing, China). β-Naphthoflavone (BNF) was purchased from Merck (Hohenbrunn, Germany). Other chemicals were commercially available and purchased as reagent grade from Sinopharm (Shanghai, China).

### 3.2. Cell Culture and Treatment

The immortalized hepatocyte cell line Fa2N-4 (Xenotech, Kansas City, KS, USA) was maintained in BEGM Bullet kit medium with 10% heat inactivated fetal bovine serum (FBS) and 1% antibiotic-antimycotic solution (Invitrogen, Carlsbad, CA, USA) at 37 °C in a humidified atmosphere of 95% air and 5% CO_2_. Fa2N-4 cells were plated in 6-well plates to achieve ~80% confluence next day. Cells were collected at 24 h after treatment with vehicle or baicalin (0–100 μM) in the presence or absence of 3ʹ,4ʹ-DMF (2 μM).

The human hepatocellular carcinoma cell line HepG2 (ATCC, Manassas, VA, USA) was maintained in DMEM containing high glucose, 10% heat-inactivated FBS, and 1% antibiotic-antimycotic solution (Invitrogen) at 37 °C in a humidified atmosphere of 95% air and 5% CO_2_. The cells were treated with baicalin (1, 10, or 50 μM) for 48 h after pGL4.10-X1/2 plasmid transfection. BNF (50 μM) was used as a positive control.

### 3.3. Animal Treatment

**Figure 7:**
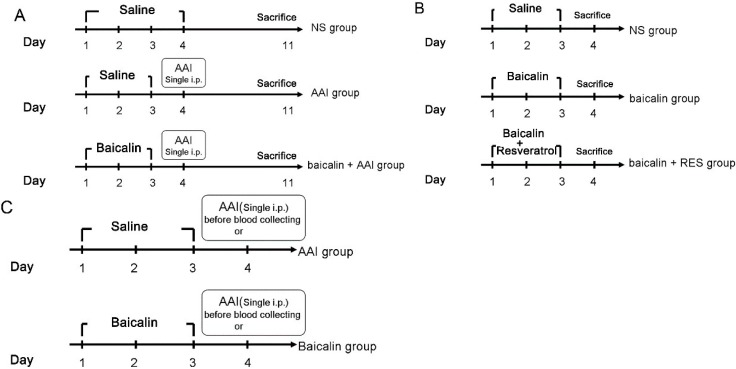
Mice were divided into 11 groups (*n* = 5 mice in each group) as follows. (**A**) Acute toxicity tests were conducted with the following groups of mice: the NS group (receiving normal saline via i.p. injection for 4 days), the AAI group (receiving 10 mg/kg AAI via i.p. injection (in warm saline) on day 4), and the baicalin (80 or 160 mg/kg) + AAI group (receiving baicalin daily for 3 days followed by a single i.p. injection of 10 mg/kg AAI on day 4); (**B**) Induction tests were conducted with the following groups of mice: The NS group (receiving normal saline via i.p. injection for 3 days), the baicalin group (receiving 80 mg/kg baicalin daily for 3 days), and the baicalin (80 mg/kg) + RES (50 mg/kg) group (receiving baicalin and RES daily for 3 days); (**C**) Pharmacokinetic assays were conducted with the following groups of mice: the NS group (receiving normal saline via i.p. injection for 3 days) with an i.p. injection of AAI (10 mg/kg) on day 4 and the baicalin group (80 or 160 mg/kg baicalin daily for 3 days) with an i.p. injection of AAI (10 mg/kg) on day 4.

Male C57BL/6 mice (6–7 weeks old, 20–22 g) were obtained from Shanghai Laboratory Animal Center. All animal experiments were approved by the Shanghai Animal Care and Use Committee (Certificate No. SCXK [Shanghai] 2002-0010). Fifty-five mice were divided into 11 groups (*n* = 5 mice each). Each group received different treatments as defined in Figure 7. Blood and liver tissues were collected and stored at −80 °C until use. Serum blood urea nitrogen (BUN) and creatinine (CRE) were measured by an automatic HITACHI Clinical Analyzer Model 7080 (Hitachi, Tokyo, Japan).

### 3.4. Histopathological Examination

Kidneys were collected at the indicated time points and fixed in 10% formalin solution before being embedded in paraffin for sectioning into 5-μm-thick sections. Sections were stained with hematoxylin and eosin (H&E) using standard pathology procedures and evaluated by a pathologist as described previously [30].

### 3.5. Determination of AAI and Its Major Metabolites in the Blood, Liver, and Kidney

For the determination of plasma AAI concentrations, blood samples were collected by tail bleeding at the indicated time points after a single intraperitoneal (i.p.) injection of 10 mg/kg AAI (Figure 1C). Blood samples (40 μL each) were collected in heparin-coated capillaries and mixed with 50 μL saline. The samples were spun at 3000× *g* for 10 min at 4 °C. Tissue samples were homogenized in saline and spun at 14,000× *g* for 10 min, and the supernatants were then mixed with 100 μg/mL (final concentration) indomethacin (internal standard, IS) and 2 volumes of methanol and spun again at 14,000× *g* for 5 min to remove precipitated proteins. Aliquots of the final supernatants were analyzed and quantified for the levels of AAI and its metabolite AAIa by high-performance liquid chromatography (HPLC), as described below.

### 3.6. HPLC Analysis

The quantification of AAI and its metabolite AAIa was carried out with a Waters 9625 HPLC system (Sunnyvale, CA, USA). For AAI, the linear ranges of the calibration curves were 0–100 μg/mL in the plasma, liver, and kidney, the regression equations were *y* = 32.186*x* + 1.1609 (*r*^2^ = 0.9992), *y* = 38.261*x* − 1.2753 (*r*^2^ = 0.9688), and *y* = 80.334*x* + 1.7436 (*r*^2^ = 0.9987), respectively, where *y* is the peak area and *x* is the concentration of the analyte. The identity of AAIa was confirmed with synthesized standards provided by Minghua Xu (Shanghai Institute of Materia Medica, Shanghai, China).

### 3.7. Real-Time PCR Analysis

Total RNA was isolated using cold Trizol reagent and first-strand cDNA was synthesized using the RT reagent kit according to the manufacturer’s protocol (Takara, Shiga, Japan). Two microliters of cDNA were used for real time PCR using TaKaRa Ex Taq RT-PCR Version 2.1 kit (TaKaRa). Gene-specific PCR primers for *CYP1A1/cyp1a1*, *CYP1A2/cyp1a2*, and *GAPDH/gapdh* are listed in Table 2, and PCR signals were detected with a DNA Engine Opticon 2 Continuous Fluorescence Detection System (Bio-Rad, Hercules, CA, USA). PCR was monitored for 45 cycles using an annealing temperature of 60 °C. At the end of the PCR cycles, melt curve analysis and 2% agar electrophoresis was performed to assess the purity of the PCR products. Negative control reactions (no template) were routinely included to monitor potential contamination of reagents. Relative amounts of *CYP1A1/cyp1a1* and *CYP1A2/cyp1a2* mRNA were normalized to that of *GAPDH/gapdh* mRNA.

**Table 2:** Primers for real-time PCR.

Primer	Sequence (5′–3′)
*CYP1A1*	Forward: CTTCCGACACTCTTCCTTCG
	Reverse: ATAGCACCATCAGGGGTGAG
*CYP1A2*	Forward: GTCACCTCAGGGAATGCTGTG
	Reverse: GTTGACAATCTTCTCCTGAGG
*GAPDH*	Forward: GGTGGTCTCCTCTGACTTCAACA
	Reverse: GTTGCTGTAGCCAAATTCGTTGT
*cyp1a1*	Forward: GACCCTTACAAGTATTTGGTCGT
	Reverse: GGTATCCAGAGCCAGTAACCT
*cyp1a2*	Forward: CCAGGTGGTGGAATCGGTG
	Reverse: TCTTAAACCTCTTGAGGGCCG
*gapdh*	Forward: GGCTACACTGAGGACCAGGTT
	Reverse: TGCTGTAGCCGTATTCATTGTC

### 3.8. Western Blot Analysis

The concentration of protein extracts from mouse hepatic microsomes and Fa2N-4 cells was determined using a BCA kit (Pierce, Rockford, IL, USA). Then protein lysates (20 μg) were separated on 10% SDA-PAGE gels followed by transfer to nitrocellulose membranes. Western blot analysis was performed as previously described [9], and the signal was detected using an ECL system (Millipore, Bedford, MA, USA). Antibodies used in this study included rabbit anti-human CYP1A1 (1:4000), mouse anti-human CYP1A2 (1:20,000), mouse anti-human β-actin (1:1000), rabbit anti-human GAPDH (1:10,000), rabbit anti-mouse cyp1a1 (1:4000), mouse anti-mouse cyp1a2 (1:20,000), and rabbit anti-CPR (1:8000).

### 3.9. Plasmid Construction and Transfection

Human CYP1A1 and CYP1A2 have a head-to-head 5′ flanking region comprising approximately 27-kb DNA segments (from +1 of the *CYP1A1* gene to +835 of the *CYP1A2* gene). The constructed plasmids contained xenobiotic response elements (XREs) from the CYP1A enhancer in transcriptional activation [23,24,31]. We chose 2 regions, one named X1 (+1 to −2867 of CYP1A1) containing 6 XRE binding locations near CYP1A1, and the other named X2 (−21,148 to −24,409 of CYP1A1) containing 1 XRE binding location near CYP1A2. Gene-specific PCR primers for X1 and X2 were as follows: X1 forward, 5′-ACCTGAGCTCGCTAGCGATCCAGAGGGAAGAGAAAA-3′ and reverse, 5′-CCGGATTGCCAAGCTTTGCACATTGATTCTTGACTC-3′; X2 forward, 5′-ACCTGAGCTCGCTAGCGGGTACCCTTGAGAAAGGAA-3′ and reverse, 5′-CCGGATTGCCAAGCTTTACCTGTAGAGGCAGGTGCT-3′. Each segment was amplified by PCR with Takara LA Taq or Primestar (Takara) and was cloned into the pGL4.10 vector (Invitrogen). All joints in the constructs were confirmed by sequencing (Sangon, Shanghai, China).

### 3.10. Luciferase Assay

HepG2 cells were seeded at a density of 1.0 × 10^5^ cells/mL in 6-well plates to achieve ~50% confluence the next day and were then transfected with pGL4.10-X1/2 plasmids using Lipofectamine 2000 (Invitrogen). At 12 h after transfection, cells were incubated with the indicated concentrations of baicalin or DMSO vehicle (0.1%) for an additional 24 h. Thereafter, cells were collected and further assayed for firefly luciferase activity, which was normalized to the activity of renilla luciferase, using the Dual-Luciferase Reporter Assay System (Promega, Madison, WI, USA) and a Biotek Synergy 4 Microplate reader (Biotek, Winooski, VT, USA). The results are presented as the ratio of luminescence of treated cell samples to control samples and are given as the mean ± SD of 3 individual transfections.

### 3.11. Statistical Analysis

The differences between each group were expressed as the mean ± SD. Statistical significance was assessed by Student’s *t*-test and one-way ANOVA followed by a Tukey *post-hoc* test. Differences were considered statistically significant if the *p*-value was less than 0.05.
